# Crystal structure and biological evaluation of 4-methyl­morpholin-4-ium 1,3-dimethyl-2,6-dioxo-5-(2,4,6-tri­nitro­phen­yl)-1,2,3,6-tetra­hydro­pyrimidin-4-olate

**DOI:** 10.1107/S2056989015010075

**Published:** 2015-05-30

**Authors:** Jeganathan Gomathi, Doraisamyraja Kalaivani

**Affiliations:** a PG and Research Department of Chemistry, Seethalakshmi Ramaswami College, Tiruchirappalli 620 002, Tamil Nadu, India

**Keywords:** crystal structure, anti­convulsant activity, hypnotic activity, barbiturate, mol­ecular salt, hydrogen bonding

## Abstract

The title mol­ecular salt, C_5_H_12_NO^+^·C_12_H_8_N_5_O_9_^−^ [common name: 4-methyl­morpholin-4-ium 1,3-dimethyl-5-(2,4,6-tri­nitro­phen­yl)barbiturate], possesses noticeable anti­convulsant and hypnotic activity. In the anion, the 1,3-di­methyl­barbituric acid ring and the symmetrically substituted tri­nitro­phenyl ring, linked *via* a C—C bond, are not coplanar but subtend an angle of 44.88 (7)°. The six-membered ring of the 4-methyl­morpholin-4-ium cation has a chair conformation. In the crystal, the cation and anion are linked *via* an N—H⋯O hydrogen bond. The cation–anion units are linked by a number of C—H⋯O hydrogen bonds, forming a three-dimensional network.

## Abstract 2

In the crystal of title mol­ecular salt, the protonated N atom of the 4-methyl­morpholin-4-ium cation forms a hydrogen bond with a carbonyl O atom of the barbiturate anion. This N—H⋯O hydrogen bond contributes to the good stability of the reported salt, which exhibits noticeable anti­convulsant and hypnotic activity.

## Chemical context   

In biological systems, pyrimidine derivatives play a significant role. Substituted barbituric acid (barbiturates) are pyrimidine derivatives which have been used as hypnotic drugs and in the treatment of epilepsy. Morpholines also have pharmacological properties and are used in organic synthesis as bases, catalysts and chiral auxiliaries (Dave & Sasaki, 2004 ▸; Mayer & List, 2006 ▸; Mossé *et al.*, 2006 ▸; Nelson & Wang, 2006 ▸; Qin & Pu, 2006 ▸). The mol­ecular salts previously synthesized in our laboratory from chloro­nitro­aromatics, barbituric acid and amines containing tertiary nitro­gen atoms possess noticeable anti­convulsant/hypnotic activity (Kalaivani & Buvaneswari, 2010 ▸; Buvaneswari & Kalaivani, 2013 ▸). In this context, we report herein on the crystal structure of a new mol­ecular salt isolated from ethano­lic solutions of 1-chloro-2,4,6-tri­nitro­benzene (TNCB), 1,3-dimethyl barbituric acid and 4-methyl­morpholine.
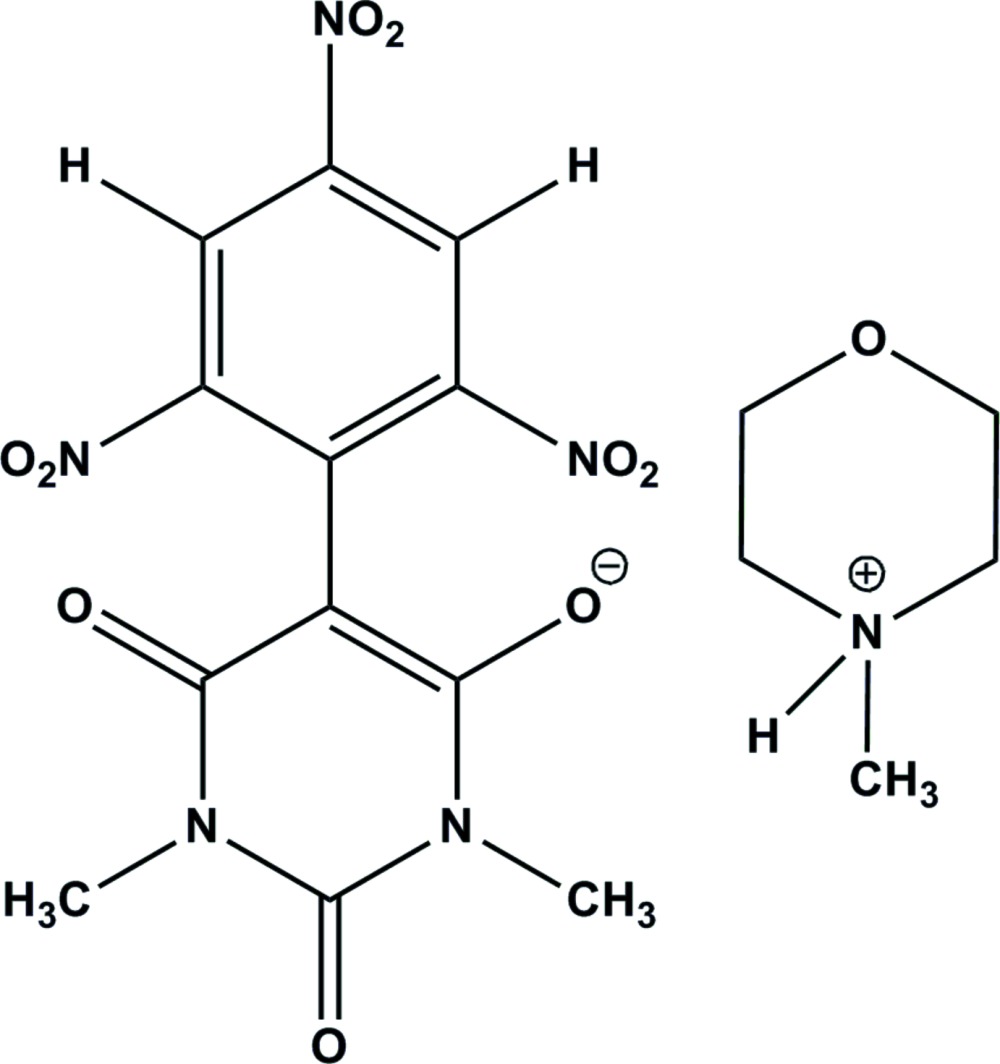


## Structural commentary   

**Figure 1:**
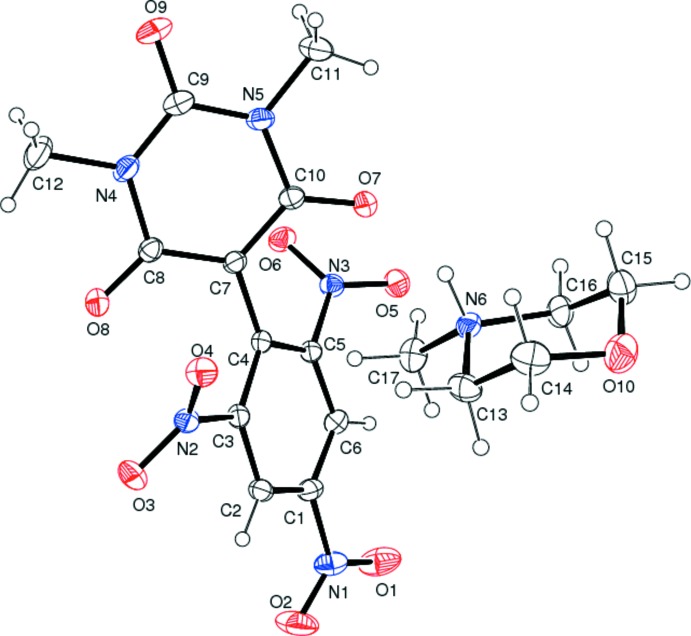
A view of the mol­ecular structure of the title mol­ecular salt, showing the atom labelling. Displacement ellipsoids are drawn at the 40% probability level.

**Figure 2:**
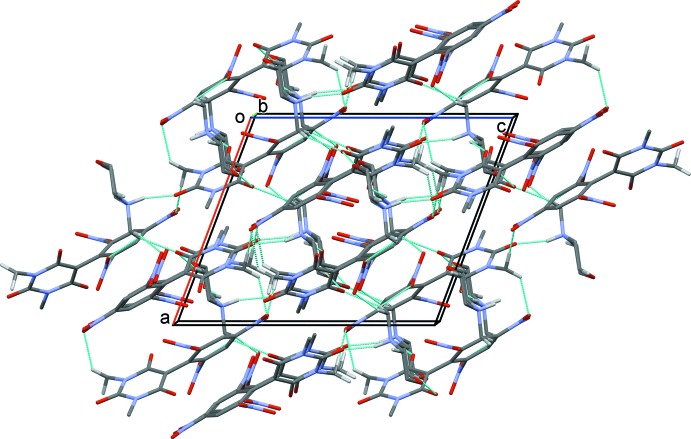
A view along the *b* axis of the crystal packing of the title mol­ecular salt. Hydrogen bonds are shown as dotted lines (see Table 1 ▸ for details).

The mol­ecular structure of the title mol­ecular salt is depicted in Fig. 1 ▸. The protonated nitro­gen atom of the *N*-methyl­morpholinium cation forms a hydrogen bond with the carbonyl group O atom of the 1,3-dimethyl-5-(2,4,6-tri­nitro­phen­yl) barbiturate anion (Table 1 ▸ and Fig. 2 ▸). This N—H⋯O hydrogen bond may well be the driving force for the formation of the title mol­ecular salt. All the bond lengths and bond angles are normal and comparable with those observed in related barbiturates (Gunaseelan & Doraisamyraja, 2014 ▸; Vaduganathan & Doraisamyraja, 2014 ▸). The six-membered morpholin-4-ium ring has a chair conformation. In the anion, the 1,3-dimethyl barbituric acid ring and the symmetrically substituted tri­nitro­phenyl ring, linked *via* the C4—C7 bond, are not co-planar but subtend an angle of 44.88 (7)°. The planes of the nitro groups substituted in the aromatic ring *ortho* with respect to the ring junction of the anion deviate to a greater extent than that of the *para* nitro group [dihedral angles of 42.66 (10) and 45.44 (9°) for the *ortho* nitro groups and 12.5 (8)° for the *para* nitro group]. Thus the *para* nitro group is more involved in delocalizing the charge of the anion than the *ortho* nitro groups, which imparts a red colour for the title mol­ecular salt.

## Supra­molecular features   

In the crystal, in addition to the N—H⋯O hydrogen bond linking the cation and anion, there are a number of C—H⋯O hydrogen bonds present, leading to the formation of a three-dimensional network, enclosing two sizable 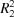
(11) and 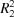
(10) ring motifs (Table 1 ▸ and Fig. 2 ▸).

## Database survey   

A search of the Cambridge Structural Database (Version 5.36, February 2015; Groom & Allen, 2014 ▸) for 5-phenyl-1,3-dimethyl barbiturates gave seven hits with various tertiary amines as cations. Two of these compounds involve 2,4-di­nitro­phenyl (CORWUD; Gunaseelan & Doraisamyraja, 2014 ▸; YAVSOF; Sridevi & Kalaivani, 2012 ▸), two involve 5-chloro-2,4-di­nitro­phenyl (DOQCUJ; Vaduganathan & Doraisamyraja, 2014 ▸), and the final three involve 2,4,6-tri­nitro­phenyl, as in the title barbiturate anion. These three compounds include the *N,N*-di­methyl­anilinium salt (JOKGIB: Babykala *et al.*, 2014 ▸), the quinolinium salt (JOKGUN: Babykala *et al.*, 2014 ▸) and the tri­ethyl­ammonium salt (LEGWIF; Rajamani & Kalaivani, 2012 ▸). In these compounds, the benzene ring is inclined to the plane of the 1,3-dimethyl barbiturate ring by 44.34, 42.88 and 46.88°, respectively, compared to 44.88 (7)° in the title salt.

## Pharmacological activity   

Epilepsy is a medical condition that produces seizures affecting a variety of mental and physical functions. Barbituric acid derivatives are potential anti-epileptic agents. The title mol­ecular salt is a derivative of 1,3-di­methyl­barbituric acid and possesses anti­convulsant activity even at low dosage (25 mg kg^−1^), inferred from the Maximal Electro Shock method on albino rats (Misra *et al.*, 1973 ▸; Kulkarni, 1999 ▸). The thera­peutic dose (100 mg kg^−1^) induces hypnosis in albino mice (Dewas, 1953 ▸) and the mol­ecular salt is non-cytotoxic on human embryonic kidney cell-HEK 293 (Mosmann, 1983 ▸).

## Synthesis and crystallization   

1-Chloro-2,4,6-tri­nitro­benzene (TNCB: 2.5 g, 0.01 mol) dissolved in 30 ml of absolute ethanol was mixed with 1,3-di­methyl­barbituric acid (1.6 g, 0.01 mol) in 30 ml of absolute ethanol. After mixing these two solutions, 3 ml of *N*-methyl­morpholine (0.03 mol) was added and the mixture was shaken vigorously for 6 to 7 h. The solution was filtered and the filtrate was kept at room temperature. After a period of four weeks, dark shiny maroon–red-coloured crystals formed from the solution. The crystals were filtered and washed with 30 ml of dry ether and recrystallized from absolute ethanol (yield: 70%; m.p.: 483 K).

## Refinement   

Crystal data, data collection and structure refinement details are summarized in Table 2 ▸. The NH H atom was located from a difference Fourier map and freely refined. The C-bound H atoms were included in calculated positions and refined as riding: C—H = 0.93–0.97 Å with *U*_iso_(H) = 1.5U_eq_(C) for methyl H atoms and 1.2*U*_eq_(C) for other H atoms.

## Supplementary Material

Crystal structure: contains datablock(s) global, I. DOI: 10.1107/S2056989015010075/su5140sup1.cif

Structure factors: contains datablock(s) I. DOI: 10.1107/S2056989015010075/su5140Isup2.hkl

Supporting information file. DOI: 10.1107/S2056989015010075/su5140Isup3.cml

CCDC reference: 1006239

Additional supporting information:  crystallographic information; 3D view; checkCIF report

## Figures and Tables

**Table 1:** Hydrogen-bond geometry (, )

*D*H*A*	*D*H	H*A*	*D* *A*	*D*H*A*
N6H6*A*O9^i^	0.90(1)	1.81(2)	2.6790(17)	162(2)
C12H12*B*O1^ii^	0.96	2.53	3.270(3)	134
C13H13*B*O8^iii^	0.97	2.42	3.046(2)	122
C15H15*A*O7^iv^	0.97	2.57	3.529(2)	169
C17H17*A*O7	0.96	2.43	3.297(2)	151
C17H17*B*O4	0.96	2.40	3.344(2)	168

Symmetry codes: (i) 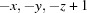; (ii) 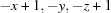; (iii) 
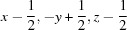
; (iv) 
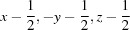
.

**Table 2:** Experimental details

Crystal data	
Chemical formula	C_5_H_12_NO^+^C_12_H_8_N_5_O_9_
*M*_r_	468.39
Crystal system, space group	Monoclinic, *P*2_1_/*n*
Temperature (K)	293
*a*, *b*, *c* ()	12.0335(2), 12.5495(2), 14.2095(3)
()	110.619(1)
*V* (^3^)	2008.38(6)
*Z*	4
Radiation type	Mo *K*
(mm^1^)	0.13
Crystal size (mm)	0.35 0.35 0.30

Data collection	
Diffractometer	Bruker Kappa APEXII CCD
Absorption correction	Multi-scan (*SADABS*; Bruker, 2004 ▸)
*T*_min_, *T*_max_	0.944, 0.979
No. of measured, independent and observed [*I* > 2(*I*)] reflections	17785, 3531, 3100
*R*_int_	0.022
(sin /)_max_ (^1^)	0.594

Refinement	
*R*[*F* ^2^ > 2(*F* ^2^)], *wR*(*F* ^2^), *S*	0.033, 0.094, 1.02
No. of reflections	3531
No. of parameters	303
No. of restraints	1
H-atom treatment	H atoms treated by a mixture of independent and constrained refinement
_max_, _min_ (e ^3^)	0.29, 0.19

Computer programs: *APEX2*, *SAINT* and *XPREP* (Bruker, 2004 ▸), *SIR92* (Altomare *et al.*, 1993 ▸), *SHELXL97* (Sheldrick, 2008 ▸), *ORTEP-3 for Windows* (Farrugia, 2012 ▸), *Mercury* (Macrae *et al.*, 2008 ▸) and *PLATON* (Spek, 2009 ▸).

## supplementary crystallographic information

### Crystal data

**Table:**

C_5_H_12_NO^+^·C_12_H_8_N_5_O_9_^−^	*F*(000) = 976
*M**_r_* = 468.39	*D*_x_ = 1.549 Mg m^−^^3^
Monoclinic, *P*2_1_/*n*	Mo *K*α radiation, λ = 0.71073 Å
Hall symbol: -P 2yn	Cell parameters from 5001 reflections
*a* = 12.0335 (2) Å	θ = 2.4–31.0°
*b* = 12.5495 (2) Å	µ = 0.13 mm^−^^1^
*c* = 14.2095 (3) Å	*T* = 293 K
β = 110.619 (1)°	Block, red
*V* = 2008.38 (6) Å^3^	0.35 × 0.35 × 0.30 mm
*Z* = 4	

### Data collection

**Table:**

Bruker Kappa APEXII CCD diffractometer	3531 independent reflections
Radiation source: fine-focus sealed tube	3100 reflections with *I* > 2σ(*I*)
Graphite monochromator	*R*_int_ = 0.022
ω and φ scan	θ_max_ = 25.0°, θ_min_ = 2.2°
Absorption correction: multi-scan (*SADABS*; Bruker, 2004)	*h* = −14→14
*T*_min_ = 0.944, *T*_max_ = 0.979	*k* = −14→14
17785 measured reflections	*l* = −14→16

### Refinement

**Table:**

Refinement on *F*^2^	Secondary atom site location: difference Fourier map
Least-squares matrix: full	Hydrogen site location: inferred from neighbouring sites
*R*[*F*^2^ > 2σ(*F*^2^)] = 0.033	H atoms treated by a mixture of independent and constrained refinement
*wR*(*F*^2^) = 0.094	*w* = 1/[σ^2^(*F*_o_^2^) + (0.0481*P*)^2^ + 0.8436*P*] where *P* = (*F*_o_^2^ + 2*F*_c_^2^)/3
*S* = 1.02	(Δ/σ)_max_ < 0.001
3531 reflections	Δρ_max_ = 0.29 e Å^−^^3^
303 parameters	Δρ_min_ = −0.19 e Å^−^^3^
1 restraint	Extinction correction: *SHELXL97* (Sheldrick, 2008), Fc^*^=kFc[1+0.001xFc^2^λ^3^/sin(2θ)]^-1/4^
Primary atom site location: structure-invariant direct methods	Extinction coefficient: 0.0055 (8)

### Special details

**Table:**

Geometry. All esds (except the esd in the dihedral angle between two l.s. planes) are estimated using the full covariance matrix. The cell esds are taken into account individually in the estimation of esds in distances, angles and torsion angles; correlations between esds in cell parameters are only used when they are defined by crystal symmetry. An approximate (isotropic) treatment of cell esds is used for estimating esds involving l.s. planes.
Refinement. Refinement of F^2^ against ALL reflections. The weighted R-factor wR and goodness of fit S are based on F^2^, conventional R-factors R are based on F, with F set to zero for negative F^2^. The threshold expression of F^2^ > 2sigma(F^2^) is used only for calculating R-factors(gt) etc. and is not relevant to the choice of reflections for refinement. R-factors based on F^2^ are statistically about twice as large as those based on F, and R- factors based on ALL data will be even larger.

### Fractional atomic coordinates and isotropic or equivalent isotropic displacement parameters (Å^2^)

**Table:**

	*x*	*y*	*z*	*U*_iso_*/*U*_eq_	
C1	0.43707 (13)	0.03855 (13)	0.25436 (11)	0.0302 (3)	
C2	0.38283 (12)	0.12808 (12)	0.27338 (11)	0.0289 (3)	
H2	0.3864	0.1927	0.2425	0.035*	
C3	0.32290 (12)	0.11897 (11)	0.33969 (10)	0.0260 (3)	
C4	0.31666 (12)	0.02532 (11)	0.39227 (10)	0.0243 (3)	
C5	0.37585 (12)	−0.06119 (11)	0.36784 (10)	0.0255 (3)	
C6	0.43254 (13)	−0.05755 (12)	0.29902 (11)	0.0293 (3)	
H6	0.4667	−0.1183	0.2833	0.035*	
C7	0.25695 (12)	0.01764 (11)	0.46538 (10)	0.0258 (3)	
C8	0.27868 (13)	0.09763 (12)	0.53973 (10)	0.0276 (3)	
C9	0.15786 (14)	−0.00393 (13)	0.61560 (12)	0.0351 (4)	
C10	0.18464 (12)	−0.07197 (11)	0.46323 (11)	0.0266 (3)	
C11	0.06174 (16)	−0.16822 (14)	0.54379 (14)	0.0426 (4)	
H11A	0.0535	−0.2146	0.4880	0.064*	
H11B	−0.0151	−0.1428	0.5396	0.064*	
H11C	0.0974	−0.2066	0.6056	0.064*	
C12	0.23622 (19)	0.16819 (15)	0.68544 (14)	0.0512 (5)	
H12A	0.2844	0.2243	0.6744	0.077*	
H12B	0.2732	0.1402	0.7521	0.077*	
H12C	0.1592	0.1958	0.6781	0.077*	
C13	−0.12719 (15)	0.08388 (12)	0.08075 (13)	0.0376 (4)	
H13A	−0.0888	0.0936	0.0317	0.045*	
H13B	−0.1145	0.1478	0.1215	0.045*	
C14	−0.25795 (15)	0.06698 (14)	0.02738 (14)	0.0455 (4)	
H14A	−0.2972	0.0616	0.0763	0.055*	
H14B	−0.2913	0.1274	−0.0159	0.055*	
C15	−0.23768 (16)	−0.11649 (14)	0.03294 (14)	0.0455 (4)	
H15A	−0.2566	−0.1813	−0.0068	0.055*	
H15B	−0.2785	−0.1189	0.0808	0.055*	
C16	−0.10612 (15)	−0.11112 (12)	0.08864 (12)	0.0371 (4)	
H16A	−0.0816	−0.1708	0.1347	0.045*	
H16B	−0.0648	−0.1161	0.0412	0.045*	
C17	0.05704 (14)	0.00254 (14)	0.19529 (13)	0.0408 (4)	
H17A	0.0886	−0.0586	0.2367	0.061*	
H17B	0.0741	0.0656	0.2362	0.061*	
H17C	0.0928	0.0083	0.1448	0.061*	
N1	0.50516 (12)	0.04650 (12)	0.18734 (11)	0.0415 (4)	
N2	0.26003 (11)	0.21678 (9)	0.34983 (9)	0.0300 (3)	
N3	0.39089 (11)	−0.16271 (10)	0.42314 (9)	0.0287 (3)	
N4	0.22370 (12)	0.08312 (10)	0.61182 (9)	0.0346 (3)	
N5	0.13701 (11)	−0.07774 (10)	0.54094 (10)	0.0320 (3)	
N6	−0.07361 (11)	−0.00932 (10)	0.14601 (10)	0.0285 (3)	
O1	0.53911 (14)	−0.03584 (12)	0.16052 (11)	0.0625 (4)	
O2	0.52504 (14)	0.13486 (12)	0.16192 (13)	0.0680 (4)	
O3	0.31154 (11)	0.30113 (9)	0.35283 (9)	0.0441 (3)	
O4	0.15911 (10)	0.20924 (9)	0.35025 (8)	0.0373 (3)	
O5	0.37978 (11)	−0.24515 (9)	0.37513 (9)	0.0412 (3)	
O6	0.41853 (10)	−0.15902 (9)	0.51449 (8)	0.0362 (3)	
O7	0.16068 (9)	−0.14407 (8)	0.40033 (8)	0.0336 (3)	
O8	0.34225 (10)	0.17649 (8)	0.54788 (8)	0.0358 (3)	
O9	0.11726 (13)	−0.01616 (11)	0.68304 (10)	0.0554 (4)	
O10	−0.27783 (11)	−0.02750 (11)	−0.03090 (9)	0.0518 (3)	
H6A	−0.1019 (14)	−0.0097 (13)	0.1966 (12)	0.033 (4)*	

### Atomic displacement parameters (Å^2^)

**Table:**

	*U*^11^	*U*^22^	*U*^33^	*U*^12^	*U*^13^	*U*^23^
C1	0.0272 (7)	0.0396 (9)	0.0254 (7)	−0.0018 (6)	0.0114 (6)	0.0027 (6)
C2	0.0295 (7)	0.0289 (8)	0.0265 (8)	−0.0053 (6)	0.0078 (6)	0.0037 (6)
C3	0.0282 (7)	0.0240 (7)	0.0246 (7)	−0.0022 (6)	0.0078 (6)	−0.0017 (6)
C4	0.0258 (7)	0.0248 (7)	0.0204 (7)	−0.0042 (5)	0.0058 (5)	−0.0020 (5)
C5	0.0282 (7)	0.0241 (7)	0.0231 (7)	−0.0021 (6)	0.0076 (6)	0.0008 (6)
C6	0.0298 (7)	0.0313 (8)	0.0271 (8)	0.0026 (6)	0.0102 (6)	−0.0003 (6)
C7	0.0319 (7)	0.0243 (7)	0.0231 (7)	0.0011 (6)	0.0121 (6)	0.0010 (6)
C8	0.0328 (8)	0.0271 (8)	0.0227 (7)	0.0037 (6)	0.0094 (6)	0.0020 (6)
C9	0.0393 (8)	0.0402 (9)	0.0310 (8)	0.0067 (7)	0.0187 (7)	0.0053 (7)
C10	0.0283 (7)	0.0267 (8)	0.0259 (7)	0.0042 (6)	0.0110 (6)	0.0040 (6)
C11	0.0441 (9)	0.0396 (9)	0.0531 (11)	−0.0032 (7)	0.0282 (8)	0.0068 (8)
C12	0.0744 (13)	0.0478 (11)	0.0387 (10)	0.0034 (9)	0.0288 (9)	−0.0116 (8)
C13	0.0439 (9)	0.0263 (8)	0.0447 (9)	0.0015 (7)	0.0184 (8)	0.0067 (7)
C14	0.0424 (10)	0.0432 (10)	0.0501 (10)	0.0077 (8)	0.0153 (8)	0.0108 (8)
C15	0.0506 (10)	0.0381 (10)	0.0482 (10)	−0.0104 (8)	0.0180 (8)	−0.0111 (8)
C16	0.0481 (9)	0.0256 (8)	0.0386 (9)	−0.0002 (7)	0.0166 (7)	−0.0072 (7)
C17	0.0376 (9)	0.0427 (10)	0.0384 (9)	0.0004 (7)	0.0088 (7)	−0.0066 (7)
N1	0.0377 (7)	0.0522 (9)	0.0408 (8)	0.0049 (7)	0.0214 (6)	0.0110 (7)
N2	0.0398 (7)	0.0241 (7)	0.0267 (7)	−0.0009 (5)	0.0127 (5)	0.0017 (5)
N3	0.0305 (6)	0.0262 (7)	0.0303 (7)	0.0015 (5)	0.0116 (5)	0.0023 (5)
N4	0.0475 (8)	0.0340 (7)	0.0266 (7)	0.0033 (6)	0.0186 (6)	−0.0028 (5)
N5	0.0367 (7)	0.0325 (7)	0.0327 (7)	−0.0003 (5)	0.0197 (6)	0.0032 (5)
N6	0.0370 (7)	0.0260 (7)	0.0256 (6)	−0.0001 (5)	0.0147 (5)	−0.0027 (5)
O1	0.0751 (10)	0.0663 (9)	0.0688 (10)	0.0253 (8)	0.0537 (8)	0.0156 (7)
O2	0.0828 (11)	0.0593 (9)	0.0880 (11)	−0.0058 (8)	0.0624 (10)	0.0168 (8)
O3	0.0609 (8)	0.0232 (6)	0.0526 (7)	−0.0081 (5)	0.0252 (6)	−0.0014 (5)
O4	0.0385 (6)	0.0355 (6)	0.0407 (7)	0.0057 (5)	0.0175 (5)	0.0028 (5)
O5	0.0580 (7)	0.0246 (6)	0.0446 (7)	0.0008 (5)	0.0224 (6)	−0.0032 (5)
O6	0.0435 (6)	0.0368 (6)	0.0264 (6)	0.0030 (5)	0.0100 (5)	0.0074 (5)
O7	0.0404 (6)	0.0290 (6)	0.0342 (6)	−0.0065 (4)	0.0166 (5)	−0.0050 (5)
O8	0.0457 (6)	0.0293 (6)	0.0325 (6)	−0.0058 (5)	0.0137 (5)	−0.0057 (5)
O9	0.0725 (9)	0.0674 (9)	0.0433 (7)	−0.0055 (7)	0.0417 (7)	−0.0029 (6)
O10	0.0483 (7)	0.0606 (9)	0.0375 (7)	−0.0019 (6)	0.0040 (6)	−0.0023 (6)

### Geometric parameters (Å, º)

**Table:**

C1—C6	1.373 (2)	C12—H12B	0.9600
C1—C2	1.373 (2)	C12—H12C	0.9600
C1—N1	1.462 (2)	C13—N6	1.491 (2)
C2—C3	1.378 (2)	C13—C14	1.502 (2)
C2—H2	0.9300	C13—H13A	0.9700
C3—C4	1.409 (2)	C13—H13B	0.9700
C3—N2	1.4753 (18)	C14—O10	1.417 (2)
C4—C5	1.407 (2)	C14—H14A	0.9700
C4—C7	1.4597 (19)	C14—H14B	0.9700
C5—C6	1.376 (2)	C15—O10	1.412 (2)
C5—N3	1.4742 (18)	C15—C16	1.502 (2)
C6—H6	0.9300	C15—H15A	0.9700
C7—C8	1.413 (2)	C15—H15B	0.9700
C7—C10	1.416 (2)	C16—N6	1.4917 (19)
C8—O8	1.2311 (18)	C16—H16A	0.9700
C8—N4	1.4134 (19)	C16—H16B	0.9700
C9—O9	1.2291 (19)	C17—N6	1.486 (2)
C9—N4	1.362 (2)	C17—H17A	0.9600
C9—N5	1.363 (2)	C17—H17B	0.9600
C10—O7	1.2325 (18)	C17—H17C	0.9600
C10—N5	1.4137 (18)	N1—O2	1.2158 (19)
C11—N5	1.462 (2)	N1—O1	1.220 (2)
C11—H11A	0.9600	N2—O3	1.2198 (16)
C11—H11B	0.9600	N2—O4	1.2201 (16)
C11—H11C	0.9600	N3—O5	1.2203 (16)
C12—N4	1.465 (2)	N3—O6	1.2221 (16)
C12—H12A	0.9600	N6—H6A	0.897 (14)
			
C6—C1—C2	121.94 (13)	H13A—C13—H13B	108.1
C6—C1—N1	118.92 (14)	O10—C14—C13	110.15 (14)
C2—C1—N1	119.11 (14)	O10—C14—H14A	109.6
C1—C2—C3	117.77 (13)	C13—C14—H14A	109.6
C1—C2—H2	121.1	O10—C14—H14B	109.6
C3—C2—H2	121.1	C13—C14—H14B	109.6
C2—C3—C4	124.77 (13)	H14A—C14—H14B	108.1
C2—C3—N2	114.05 (12)	O10—C15—C16	111.21 (14)
C4—C3—N2	121.16 (12)	O10—C15—H15A	109.4
C5—C4—C3	112.75 (12)	C16—C15—H15A	109.4
C5—C4—C7	122.91 (12)	O10—C15—H15B	109.4
C3—C4—C7	124.33 (12)	C16—C15—H15B	109.4
C6—C5—C4	124.77 (13)	H15A—C15—H15B	108.0
C6—C5—N3	114.14 (12)	N6—C16—C15	110.58 (13)
C4—C5—N3	120.87 (12)	N6—C16—H16A	109.5
C1—C6—C5	117.90 (14)	C15—C16—H16A	109.5
C1—C6—H6	121.0	N6—C16—H16B	109.5
C5—C6—H6	121.0	C15—C16—H16B	109.5
C8—C7—C10	122.06 (13)	H16A—C16—H16B	108.1
C8—C7—C4	118.51 (12)	N6—C17—H17A	109.5
C10—C7—C4	119.34 (12)	N6—C17—H17B	109.5
O8—C8—C7	125.91 (13)	H17A—C17—H17B	109.5
O8—C8—N4	117.99 (13)	N6—C17—H17C	109.5
C7—C8—N4	116.08 (13)	H17A—C17—H17C	109.5
O9—C9—N4	121.84 (15)	H17B—C17—H17C	109.5
O9—C9—N5	120.48 (15)	O2—N1—O1	123.84 (14)
N4—C9—N5	117.69 (13)	O2—N1—C1	118.02 (15)
O7—C10—N5	118.24 (13)	O1—N1—C1	118.14 (14)
O7—C10—C7	125.72 (13)	O3—N2—O4	124.16 (13)
N5—C10—C7	116.04 (13)	O3—N2—C3	116.98 (12)
N5—C11—H11A	109.5	O4—N2—C3	118.78 (12)
N5—C11—H11B	109.5	O5—N3—O6	124.13 (12)
H11A—C11—H11B	109.5	O5—N3—C5	117.77 (12)
N5—C11—H11C	109.5	O6—N3—C5	118.02 (12)
H11A—C11—H11C	109.5	C9—N4—C8	124.01 (13)
H11B—C11—H11C	109.5	C9—N4—C12	118.14 (14)
N4—C12—H12A	109.5	C8—N4—C12	117.84 (14)
N4—C12—H12B	109.5	C9—N5—C10	123.98 (13)
H12A—C12—H12B	109.5	C9—N5—C11	116.88 (13)
N4—C12—H12C	109.5	C10—N5—C11	119.14 (13)
H12A—C12—H12C	109.5	C17—N6—C13	111.63 (12)
H12B—C12—H12C	109.5	C17—N6—C16	111.94 (12)
N6—C13—C14	110.49 (13)	C13—N6—C16	111.05 (12)
N6—C13—H13A	109.6	C17—N6—H6A	105.1 (11)
C14—C13—H13A	109.6	C13—N6—H6A	107.4 (11)
N6—C13—H13B	109.6	C16—N6—H6A	109.4 (11)
C14—C13—H13B	109.6	C15—O10—C14	109.69 (13)
			
C6—C1—C2—C3	0.1 (2)	C6—C1—N1—O1	11.8 (2)
N1—C1—C2—C3	−177.70 (13)	C2—C1—N1—O1	−170.27 (15)
C1—C2—C3—C4	2.4 (2)	C2—C3—N2—O3	−40.93 (17)
C1—C2—C3—N2	−175.69 (13)	C4—C3—N2—O3	140.92 (14)
C2—C3—C4—C5	−1.9 (2)	C2—C3—N2—O4	135.91 (13)
N2—C3—C4—C5	176.08 (12)	C4—C3—N2—O4	−42.23 (19)
C2—C3—C4—C7	177.42 (13)	C6—C5—N3—O5	−44.54 (17)
N2—C3—C4—C7	−4.6 (2)	C4—C5—N3—O5	140.67 (13)
C3—C4—C5—C6	−1.1 (2)	C6—C5—N3—O6	132.46 (13)
C7—C4—C5—C6	179.58 (13)	C4—C5—N3—O6	−42.33 (18)
C3—C4—C5—N3	173.08 (12)	O9—C9—N4—C8	175.76 (15)
C7—C4—C5—N3	−6.2 (2)	N5—C9—N4—C8	−4.8 (2)
C2—C1—C6—C5	−2.8 (2)	O9—C9—N4—C12	−5.7 (2)
N1—C1—C6—C5	174.99 (13)	N5—C9—N4—C12	173.71 (15)
C4—C5—C6—C1	3.4 (2)	O8—C8—N4—C9	−175.43 (14)
N3—C5—C6—C1	−171.15 (13)	C7—C8—N4—C9	3.2 (2)
C5—C4—C7—C8	132.29 (14)	O8—C8—N4—C12	6.0 (2)
C3—C4—C7—C8	−46.92 (19)	C7—C8—N4—C12	−175.31 (14)
C5—C4—C7—C10	−44.30 (19)	O9—C9—N5—C10	−177.15 (15)
C3—C4—C7—C10	136.49 (14)	N4—C9—N5—C10	3.4 (2)
C10—C7—C8—O8	178.39 (14)	O9—C9—N5—C11	2.1 (2)
C4—C7—C8—O8	1.9 (2)	N4—C9—N5—C11	−177.34 (14)
C10—C7—C8—N4	−0.2 (2)	O7—C10—N5—C9	179.21 (14)
C4—C7—C8—N4	−176.65 (12)	C7—C10—N5—C9	−0.6 (2)
C8—C7—C10—O7	179.15 (14)	O7—C10—N5—C11	0.0 (2)
C4—C7—C10—O7	−4.4 (2)	C7—C10—N5—C11	−179.82 (13)
C8—C7—C10—N5	−1.0 (2)	C14—C13—N6—C17	176.77 (14)
C4—C7—C10—N5	175.41 (12)	C14—C13—N6—C16	51.09 (18)
N6—C13—C14—O10	−57.98 (18)	C15—C16—N6—C17	−175.33 (14)
O10—C15—C16—N6	55.94 (19)	C15—C16—N6—C13	−49.82 (17)
C6—C1—N1—O2	−167.86 (16)	C16—C15—O10—C14	−63.10 (18)
C2—C1—N1—O2	10.0 (2)	C13—C14—O10—C15	63.87 (18)

### Hydrogen-bond geometry (Å, º)

**Table:**

*D*—H···*A*	*D*—H	H···*A*	*D*···*A*	*D*—H···*A*
N6—H6*A*···O9^i^	0.90 (1)	1.81 (2)	2.6790 (17)	162 (2)
C12—H12*B*···O1^ii^	0.96	2.53	3.270 (3)	134
C13—H13*B*···O8^iii^	0.97	2.42	3.046 (2)	122
C15—H15*A*···O7^iv^	0.97	2.57	3.529 (2)	169
C17—H17*A*···O7	0.96	2.43	3.297 (2)	151
C17—H17*B*···O4	0.96	2.40	3.344 (2)	168

Symmetry codes: (i) −*x*, −*y*, −*z*+1; (ii) −*x*+1, −*y*, −*z*+1; (iii) *x*−1/2, −*y*+1/2, *z*−1/2; (iv) *x*−1/2, −*y*−1/2, *z*−1/2.
